# Open LED Illuminator: A Simple and Inexpensive LED Illuminator for Fast Multicolor Particle Tracking in Neurons

**DOI:** 10.1371/journal.pone.0143547

**Published:** 2015-11-23

**Authors:** Jens B. Bosse, Nikhila S. Tanneti, Ian B. Hogue, Lynn W. Enquist

**Affiliations:** 1 Department of Molecular Biology, Princeton University, Princeton, 08544, New Jersey, United States of America; 2 Princeton Neuroscience Institute, Princeton University, Princeton, 08544, New Jersey, United States of America; The Beatson Institute for Cancer Research, UNITED KINGDOM

## Abstract

Dual-color live cell fluorescence microscopy of fast intracellular trafficking processes, such as axonal transport, requires rapid switching of illumination channels. Typical broad-spectrum sources necessitate the use of mechanical filter switching, which introduces delays between acquisition of different fluorescence channels, impeding the interpretation and quantification of highly dynamic processes. Light Emitting Diodes (LEDs), however, allow modulation of excitation light in microseconds. Here we provide a step-by-step protocol to enable any scientist to build a research-grade LED illuminator for live cell microscopy, even without prior experience with electronics or optics. We quantify and compare components, discuss our design considerations, and demonstrate the performance of our LED illuminator by imaging axonal transport of herpes virus particles with high temporal resolution.

## Introduction

The advent of inexpensive and easy-to-use microcontrollers, coupled with the rapid progress in 3D printing technology has fueled the so-called “maker movement”, a fast growing do-it-yourself community that develops and shares open-source hardware designs ranging from simple flashlights to sophisticated robots. As software and hardware designs are shared freely, it is now straightforward to build custom machinery from basic hardware building blocks with a significant reduction in time and resources. Projects like OpenSPIM [1] and Open Labware [2] exemplify this movement in the scientific community, which gives scientists outside of engineering fields the ability to quickly build custom research equipment, tailored to their needs, and at much reduced cost.

Epifluorescence microscopy is a simple and effective way to image particle and organelle motility in cultured neurons. Traditional fluorescence illuminators, however, produce broad-spectrum light and need some kind of channel separation. This is often done by using motorized filter wheels for multicolor imaging with black and white cameras. As fast transport in axons generally averages 1–2 μm/sec and can reach much higher instantaneous velocities, a single two-color particle can artifactually appear to be two adjacent single-color particles due to particle movement during slow mechanical filter switching. This artifact can pose problems for interpreting colocalization, quantifying particle fluorescence, and tracking individual particles in crowded cellular environments.

Alternative light sources include laser diodes which can be modulated rapidly; however, they can be expensive and require a more sophisticated optical design due to the coherent nature of laser light. Light Emitting Diodes (LEDs) on the other hand, are inexpensive, non-coherent light sources whose output can be modulated in microseconds. Combining several LEDs with multiband filters completely eliminates the need for mechanical filter switching, making them very useful for live cell imaging.

Here, we describe a simple, open hardware design to build a high-performance dual-color LED illuminator, readily available stock hardware, for less than $3000 USD. The procedure we detail enables any scientist to build such a system, even if they have no prior experience in electronics or optics. The resulting system is capable of switching between individual fluorescence channels in less than a millisecond. As proof of principle, we characterize the system’s performance and demonstrate its usefulness by tracking fluorescent virus particles in live neuron cultures.

## Materials and Methods

### Assembly of the LED illuminator

All parts used in this work are listed in S1 Table.

### Quantification of LED brightness

LED brightness was measured by coupling the LED illuminator described in this work including a Thorlabs DMLP505R dichroic mirror to combine channels onto the Koehler fluorescence illuminator unit of an Nikon Ti inverted microscope stand equipped with a Nikon Plan Fluor ELWD 20x/0.45 Ph1 DM objective. Chroma red and green fluorescent plastic slides (Chroma 92001) were used as samples. Images were acquired with a Photometrics Coolsnap ES^2^ CCD camera. A Chroma ET-EGFP/mCherry set (Chroma 59022) was used for LED illumination. For Arc-lamp illumination the dual bandpass excitation filter was exchanged with the equivalent single bandpass excitation filters (ET470/40x and ET572/35x). An ND8 filter (12.5% transmittance) was used to attenuate the excitation light and images were taken at 1 ms exposure time. All used LEDs are listed in S4 Table together with their drive currents. An Excelitas X-Cite 120 mercury vapor short arc lamp was used for comparison. The bulb had been used for less than 50 hours with limited on/off cycles. At least 10 images were taken from different sample positions and their mean value was determined in Fiji [3].

### Quantification of the effects of critical and Koehler illumination

Brightness and illumination flatness in critical and Koehler illumination modes were measured on a custom-built epifluorescence microscope using Nikon Plan Fluor ELWD 20x/0.45 Ph1 DM objective, a Nikon 200 mm tube lens and a Chroma ET-EGFP/mCherry set (Chroma 59022) using a Thorlabs DCU3240 CMOS camera.

All diaphragms and the extra elements to generate conjugate planes were omitted to reduce path length and increase light throughput. LEDs were either collimated and projected directly onto the objective back focal plane (critical illumination) or a 150 mm achromatic lens was used to focus the light onto the objectives back focal plane (Koehler illumination). Lumileds Luxeon Rebel blue (470 nm LED) or Luxeon Rebel PC amber (595 nm LED) were used and driven at 1000 mA. Images were acquired either for 0.2 ms with green Chroma fluorescent plastic slides (470 nm LED) or for 1 ms (595nm LED) with red Chroma fluorescent plastic slides.

### Live cell imaging of axonal transport

Embryonic superior cervical ganglion (SCG) neurons were obtained from Sprague-Dawley rats in strict accordance with the Guide for the Care and Use of Laboratory Animals of the National Institutes of Health and the Princeton University Institutional Animal Care and Use Committee (protocol number 1947–13). SCG neurons were extracted and cultured in modified Campenot tri-chambers, as previously described [4]. Neurons in the soma compartment were infected with approximately 5x10^6^ plaque forming units of PRV 137, which expresses gM-EGFP/mRFP-VP26 [5]. 10 hours post infection viral particle trafficking was imaged in axons penetrating into the middle and neurite compartments.

For LED illumination, the LED illuminator housing an Oslon SSL 80 470 nm LED (Thorlabs M470D2) and a Lumileds Luxeon Rebel PC amber (Thorlabs M595D2 or Luxeonstar) was coupled onto the Koehler fluorescence illuminator unit of an Nikon Ti inverted microscope stand equipped with a Nikon Plan Apo VC 100x/1.40 Oil DIC N2 objective. Images were acquired with an Andor iXON3 897 EMCCD camera. An ND8 filter (12.5% transmittance) was used to attenuate all excitation light of Arc lamp illumination while an ND 10 filter (10.0% transmittance) was used directly in front of the blue LED. Images were taken at 50 ms exposure time with an EM gain at 90. Filter changes during metal halide lamp illumination were done using a Prior Proscan II controller and two Prior motorized filter wheels.

## Results

Several good introductions to the use of LEDs for microscopy can be found elsewhere [6–8]. Here we provide a practical guide focused on the use of LEDs for fast, dual-color live cell fluorescence microscopy.

The net light output and beam quality of a LED illuminator are influenced by several important factors. In the following sections we will discuss our design considerations, hardware choices, and present a detailed assembly protocol.

### Design considerations

#### The ideal light source for fluorescence microscopy

The ideal light source for fluorescence microscopy is a point source, non-coherent, narrow bandwidth, has an easily modulated and a stable power output. Traditionally used arc lamps only partially fulfill these requirements, as they are typically broad range sources that cannot be modulated. LED emitters on the other hand have some unique features that make them a potentially better light source. Most importantly, “single color” LEDs are available that have a relatively narrow bandwidth. Together with their ability to be rapidly modulated and dimmed, these LEDs are the ideal candidates as single channel light sources for rapid imaging. However emitter size and radiance profile can be a problem, and will be discussed below.

#### Channel separation

In multicolor fluorescence imaging, distinct spectral emission bands are used to encode separate channels. There are different options to record these channels separately. One often used method is the temporal separation of individual channels. Here, temporally separated narrow bands of excitation light are used to excite different fluorophores consecutively, and their emission is recorded on a black and white camera. In combination with typically used broad-spectrum light sources, this method has the downside that relatively slow motorized filter wheels are needed to switch channels, leading to motion artifacts in live cell microscopy.

An alternative is to use a color camera and multiband filter sets, which enable the simultaneous acquisition of up to three spectral bands (red, green, blue). However color cameras suffer from lower sensitivity than black and white cameras and spectral separation of channels might be problematic depending on the choice of fluorophores.

Another option is the spatial separation of channels by using a dichroic mirror and projecting both channels onto different areas of the same camera. While this method allows very fast dual or even multicolor-imaging without mechanical filter wheel switching, it reduces the amount of sensor area effectively used for the final image. In addition, strongly differing channel brightness might be problematic and optimization with neutral density filters might be needed. This problem can be omitted by using two cameras each imaging one channel, however this option might be cost-prohibitive.

Due to the above reasons, we believe that temporal separation of channels using individual, spectrally well-separated LED sources is right now the most cost-effective and flexible way for multicolor-fluorescence imaging. With this method, slow filter-wheels are omitted and emitters can even be dimmed electronically and individually, which further improves utility.

#### Fluorescence Filters

The choice of fluorescence filters ultimately determines the fluorophores that can be used. For dual-color live cell microscopy it is best to use spectrally well separated fluorescent protein pairs, such as green and red fluorescent proteins. A compatible full multiband filter set, consisting of a multiband dichroic mirror and multiband emission filter, will allow rapid fluorescence channel switching by LEDs without the need for mechanical filter movement. But because multiband emission and excitation filters are used (in contrast to Pinkel and Sedat filter sets, with either single band exciters, or both single band exciters and single band emitter filters, respectively), it is very important to control for bleed through. A second dichroic mirror is required to combine two LEDs into the optical path. For all filters, special attention should be given to match their transmission and reflection spectra to the intrinsic spectra of the LEDs. This is not always possible, but some optical filter manufacturers now sell specialized LED filter sets that are optimized to the spectra of certain commercial LED sources. Care should be also taken if phosphor-converted LEDs are used, as they may have emission spectra with long tails that can lead to bleed through.

#### LEDs

Several factors critical for choosing the LEDs are discussed here in their order of importance. The first factor to consider is spectrum, or color, produced by an LED. As described in the above section, LED spectra should be matched to that of the available fluorophores and optical filters.

**Fig 1:**
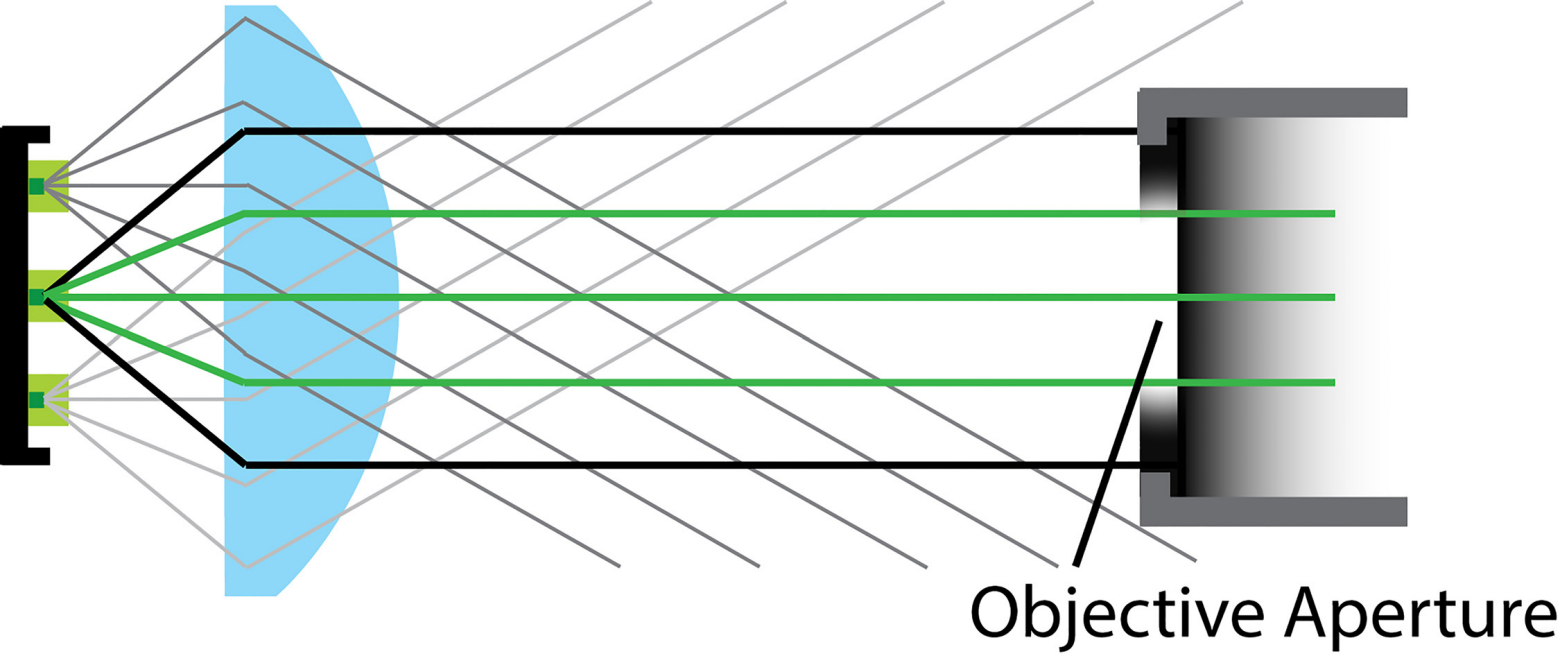
Efficiency of light projection is dependent on the LEDs etendue. A lens (blue) is used to collimate the light from an LED with multiple emitters (green squares). While light from the central emitter (green rays) is projected onto the objective aperture, light from off-center LEDs does not reach the objective aperture (grey rays). In addition, outer beam angles produce a wider beam, which also cannot be directed into the objective aperture (black rays).

The next important factor to consider is “etendue”, a term referring to how “spread out” is the generated light in terms of area and angle. For LEDs, the entendue is determined by the angle of the emitted light beam and the size of the light-emitting chip. As shown in Fig 1, with greater etendue, the less of the light produced can be guided to the microscope objective. A good example can be found in reference [9]: Assuming an angle of 24° for the objective, a 4.25 mm LED chip with 180° beam angle would transmit only 4% of its light into the objective, while a LED of 1 mm length and 120° beam angle would transmit 75% of its light. Some manufacturers produce large, high-density LED assemblies that can be several cm in size. These assemblies can produce enormous amounts of light (and heat!, see below), but very little of the light can be effectively used. Generally, the smaller the emitter surface and smaller the beam angle, the more light can be used.

Efficient cooling is also of great importance as LED efficiency decreases with increasing temperature. In general, passive cooling using large aluminum or copper heatsinks is better suited for microscopy than using fans which can cause vibrations into the imaging system.

There are, however, specialized LED optics available that rely on total internal reflection to form a more collimated beam (e.g. light pipes). Another option is to use elliptical mirrors to focus the emitted light. However, in our experience these parts either are expensive or of inferior optical quality. We therefore recommend using LEDs with small emitters and narrow beam angles.

The next consideration is the total light output of the LED. Output is often given in lumens, which can be misleading because measurements in lumens factor in the sensitivity of the human eye to different wavelengths of light. A function to convert lumens to approximate wattage can be used to compare LEDs of different wavelengths. Some third-party suppliers also provide power measurements in mW [10], which can be very helpful in comparing LEDs.

The final point to consider is LED binning. Most manufacturers bin their production into several batches based on spectral width and optical output. The differences between the highest and lowest output bins can be as large as 50%, so care should be taken to select not only the right model, but also the appropriate bin. Depending on the manufacturer and model it might even be necessary to compare several LEDs from the same bin as individual LEDs can vary in their output by up to a factor of three [11].

#### Comparison of LEDs for EGFP/mCherry excitation

Here, we describe a simple two-color illuminator for the excitation of EGFP and mCherry fluorescent proteins, or fluorescent proteins and fluorophores with similar excitation spectra. EGFP has an excitation peak at 488 nm and mCherry peaks at about 586 nm. Both proteins have relative broad excitation peaks, which facilitates finding compatible filter and LED combinations.

The progress in LED development has been rapid and “Haitz’s law” [12] predicts a 10-20x increase in light output per decade. Therefore, while we recommend best choices at present, the reader is advised to reevaluate our recommendation as new and more powerful LEDs come to market.

**Fig 2:**
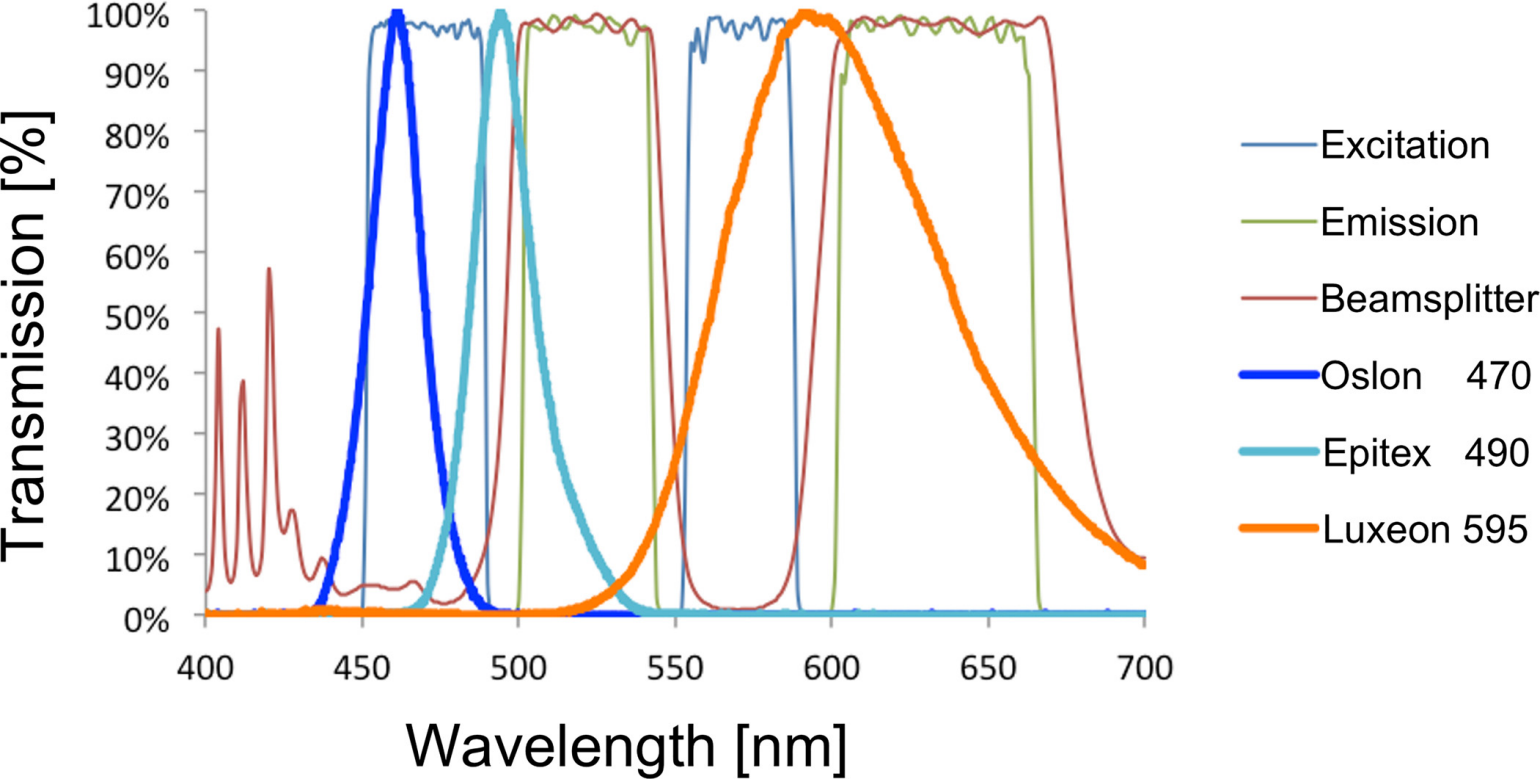
Filterset choice determines LED choice. The excitation (blue, thin line), emission (red, thin line) and dichroic filter properties (green, thin line) for the Chroma ET-EGFP/mCherry set (Chroma 59022) provided by Chroma [13] are plotted and overlaid with the spectra of the Oslon SSL 80 470nm LED (Oslon 470, blue, thick line), the Epitex SMBB490-1100-02 490 nm LED (Epitex 490, cyan, thick line) and the Luxeon PC amber 595 nm (Luxeon 595 amber, thick line) provided by Thorlabs [14]. The 470 nm LED spectrum fits well with the first excitation of the filter set, while the 490 nm LED light, although more ideal for the excitation of EGFP is largely blocked by the excitation filter. The phosphor-converted 595 LED spectrum is broad and only parts of the emitted spectrum can be used.

Current LED technology is very efficient in the blue (450–470 nm) range but less optimal in the cyan range (490 nm) that is typically used for EGFP excitation. We therefore recommend choosing bluer LEDs, centered around 470 nm. These LEDs still excite GFP well due to their broad spectrum (≈460–480 nm) while being about 4x brighter than LEDs in the 490 nm range. Similarly, LED technology is very efficient in producing red light in the 620–660 nm range, but needs improvement in the yellow-green to orange range (550–600 nm). In this latter range, manufacturers often couple LEDs to phosphors, which produce broad spectra but with lower peak power. For these reasons, we used the Chroma ET-EGFP/mCherry filter set (Chroma 59022). As illustrated in Fig 2, this filter incorporates a 470/40 bandgap that works well with LEDs with outputs centered around 470 nm and a second bandgap of about 572/35 that captures some parts of the phosphor-converted spectrum in the yellow range. Finally, to combine both LEDs, we used a dichroic mirror with a longer cutoff wavelength (505 nm) (Thorlabs DMLP505R) to use as much blue light as possible.

**Fig 3:**
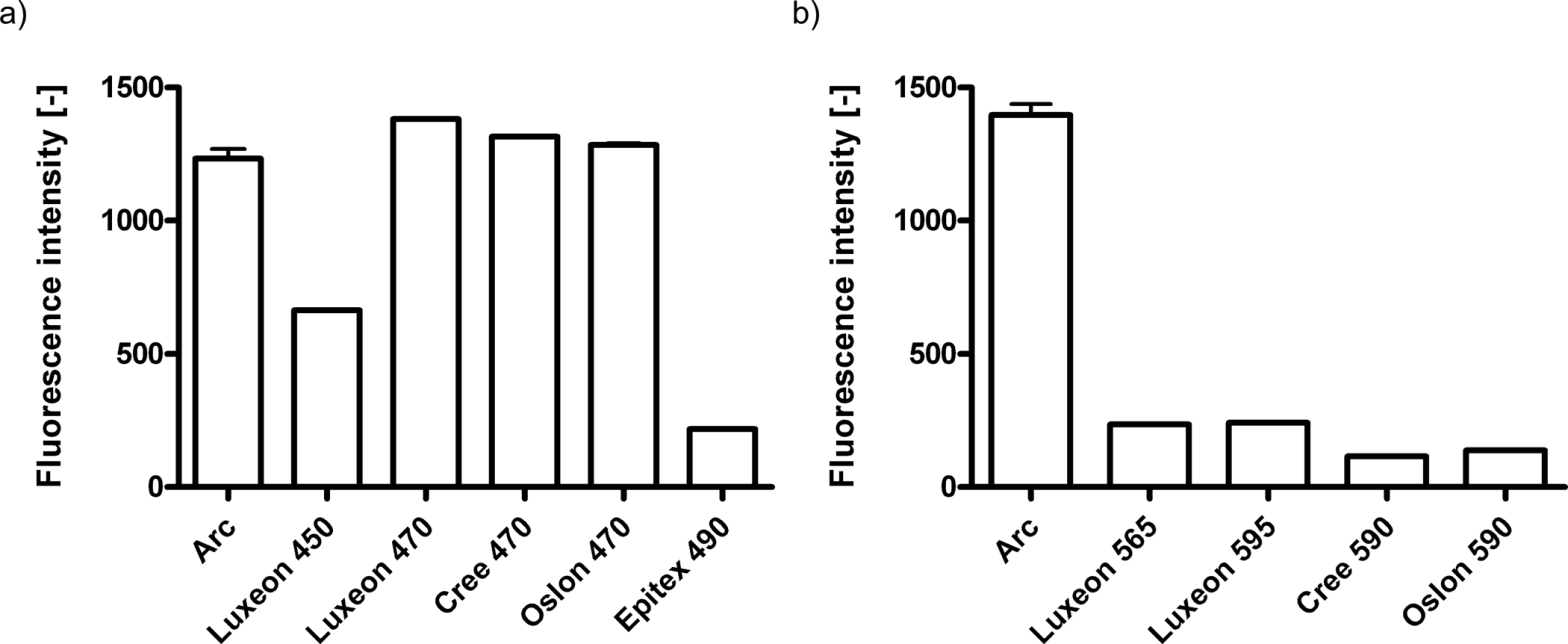
Comparison of Arc lamp and LED illumination intensities. The output of several LEDs was compared to the output of a 120W Mercury Vapor Short Arc lamp (Excelitas X-cite 120) by using Chroma fluorescent plastic slides as samples (see Materials and Methods for details). a) Comparison of blue LEDs to Arc lamp excitation (Arc). LEDs tested were Lumileds Luxeon Rebel royal blue (Luxeon 450), Lumileds Luxeon Rebel blue (Luxeon 470), Cree XT-E2 blue (Cree 470), Oslon SSL 80 470 nm (Oslon 470) and Epitex SMBB490-1100-02 490 nm (Epitex 490). The LEDs centered around 470 nm performed better as they better matched the filter set used. b) Comparison of red LEDs to Arc lamp excitation (Arc). LEDs tested were Lumileds Luxeon Rebel lime (Luxeon 565), Lumileds Luxeon Rebel PC amber (Luxeon 595), Cree XT-E2 PC amber (Cree 590) and Oslon SSL 80 590 nm (Oslon 590). The best performing LEDs in the amber range were about 6 fold less efficient than an Arc lamp in this spectral region. Shown are the averages and standard deviations of the mean intensities of at least 10 individual images.

To find the optimal LED for this filter combination, we measured the intensity of a variety of LEDs, and compared them to a 120W metal halide lamp (Excelitas X-cite 120). As the metal halide lamp is a broad excitation source, single band excitation filters had to be used instead of the dual band excitation filter used for LED illumination. These single band excitation filters matched the respective dual band filters very closely (see Material and Methods for details). All other filters were kept unchanged and imaging conditions were also kept the same. As shown in Fig 3A for blue light and in Fig 3B for orange light (see S2 and S3 Tables for raw data), LEDs with spectra that do not match the filter set, performed poorly (see also Fig 2), even if their beam angle was much more favorable [compare Epitex SMBB490-1100-02 490 nm LED (22°) with Luxeon Rebel blue 470 nm (120°)]. Interestingly, the Oslon SSL 80 470nm LED, with a beam angle of 80° did not perform better than the Luxeon Rebel blue 470 nm LED with an angle of 120° emitting in the same range. Comparison to a light guide coupled 120W metal halide lamp (Excelitas X-Cite 120) showed that the three best performing LEDs (Luxeon Rebel blue 470nm, Oslon SSL 80 470 nm and Cree X-TE blue 470 nm) were equal or slightly brighter, arguing that these three LEDs are good choices for replacing arc lamps in this spectral range.

In contrast, none of the yellow-green to amber LEDs tested were as bright as the metal halide lamp output (Fig 3B). The two best performers (Luxeon Rebel lime 565 nm and Luxeon Rebel 595 nm) were still about 6-fold dimmer than the metal halide lamp. The most significant problem appears to be the broad spectrum and low peak power for the phosphor-converted LEDs in this spectral region. Amber LEDs that do not rely on phosphor conversion do exist (e.g. Luxeon Rebel amber or Cree XT-E amber). These LEDs have a narrower spectrum and up to 2x higher peak powers. Unfortunately, their peak wavelengths are 10–20 nm too long for all of the commercial dual-band filters available to us. In addition, the spectral output of typical arc lamps is weakest in the blue region while very strong in the green-yellow-amber region, further making it difficult to match the illumination intensity. For this design we recommend the Lumileds Luxeon Rebel blue (470 nm) and Lumileds Luxeon Rebel PC amber (595 nm). All tested LEDs are summarized in S4 Table.

#### Illumination modes

**Fig 4:**
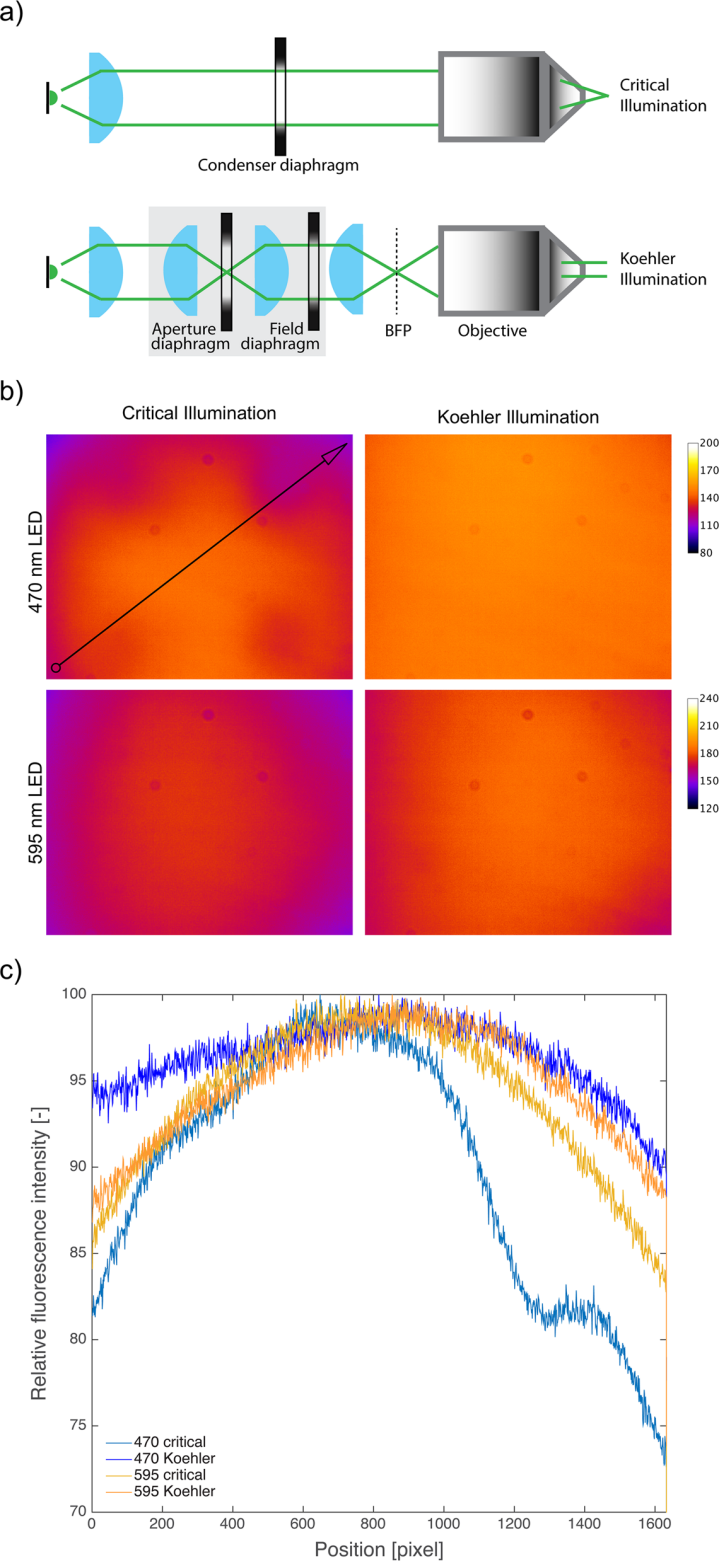
Koehler illumination results in superior illumination evenness. a) Schematic of critical (top) and Koehler modes. Images were inspired by [15]. In critical illumination, the LED light (green) is collimated using a collimation lens (blue) and projected onto the objective. The objective focuses the light, resulting in an image of the LED at the sample plane. In Koehler illumination mode, the collimated light gets focused onto the back focal plane (BFP) of the objective using a second lens (right most lens, blue). This way, the objective projects a collimated cone of light onto the sample plane, resulting in better illumination flatness. A set of two more lenses can be used to generate conjugate planes that than can be regulated by diaphragms to adjust the brightness, contrast, resolution and depth of field of the image (Aperture diaphragm) and the illuminated field of view (Field diaphragm) (grey box). In critical illumination with infinite conjugate plate, an Aperture diaphragm can be used to regulate brightness, contrast, resolution and depth of field. b) Brightness and illumination flatness in critical and Koehler illumination modes were measured on a custom-built epifluorescence microscope (see Materials and Methods for details). Representative images are shown and intensities are coded as colors as indicated in the legend and can be compared between critical and Koehler modes but not between LEDs as different exposure times were used. Koehler illumination showed a better field flatness and slightly increase in intensity (≈ 1.3–29.9% depending on image position for the 470 nm LED and about 2.8–12.96% for the 595 nm LED (see also S5 Table)). c) Comparison of fluorescence intensity distributions. At least three images were taken and the fluorescent plastic slide was moved between images. Images were analyzed in Fiji [3] by drawing a diagonal line ROI and intensities along that line were measured using the “plot profile” tool (black arrow). Resulting intensity values were imported into Matlab (Mathworks), the mean was calculated for three images each and means were normalized to their individual maxima and plotted as a line plot depicting the degree of illumination differences over the measured diagonal. Critical illumination with directly emitting LEDs (470 nm LED) lead to uneven illumination, while the evenness with the phosphor-converted LED (595 nm LED) was only slightly worse than with Koehler illumination.

There are two primary ways to collect and project the LED light: critical illumination and Koehler illumination. A good online resource can be found in reference [15]. Critical illumination for inverted fluorescence microscopy can be achieved simply by placing a single collimator lens in front of the LED. The microscope objective serves as the condenser (Fig 4A), thus eliminating the need for other optical elements. An iris (i.e. aperture diaphragm) can be included to vary the maximal angle of light rays that hit the objective (i.e. numerical aperture, NA), thereby controlling the image contrast and resolution. This option is efficient and easy. However, because an image of the LED is projected onto the sample, the field of illumination is not uniform. A diffuser plate can be used to smooth out the LED image but we found that this strongly reduced the illumination intensity.

Koehler illumination allows for homogenous projection of light onto the sample by focusing the LED image onto the back focal plane of the objective, while the LED light is focused to infinity at the sample plane. In addition, a second iris at the sample conjugate plane can be added (i.e. field iris), which allows the adjustment of the illumination area and reduces phototoxicity in areas not imaged. We used a 150 mm achromatic lens to focus onto the objectives back focal plane. Together with the 20 mm condenser lens this results in a 7.5x magnification of the LED chip. Both, the Luxeon and Oslon emitters are about 1 mm in length such that the resulting image is about 7.5 mm long. Ideally this image should fill the exit pupil of the objective which can be estimated using following formula: 2*focal-length-of-the-objective*NA. In case of a 20x, 0.45 NA Nikon objective (with a 200 mm tube length resulting in 200mm/20 = 10 mm focal length) the image should be ideally 9 mm in size to fill the exit pupil. We therefore slightly underfilled the objective.

We compared critical illumination to Koehler illumination using fluorescent plastic slides (Fig 4B). First, we measured the intensity of a full Koehler setup, but the extra path length and the extra optical elements strongly reduced illumination intensity. As both the aperture and field diaphragms are often left fully open for epifluorescence microscopy, we omitted the extra optical elements used to adjust the field and aperture plane (grey box in Fig 4A). We found that critical illumination was problematic for LEDs with a patterned chip surface like the Luxeon 470 (Fig 4B and 4C). Other LEDs may also have wires on their surface (e.g. Oslon SSL 80 470 nm) and the images of these wires can be projected into the sample. In these cases, Koehler illumination works best. For LEDs without much structure, like the phosphor-converted Luxeon Rebel PC amber, Koehler illumination did not produce more illumination uniformity, but did produce slightly more intensity (≈ 1.3–29.9% depending on image position for the 470 nm LED and about 2.8–12.96% for the 595 nm LED, see also S5 Table for raw data).

### Assembly

#### LED assembly

LEDs typically are sold on printed circuit boards (PCBs) with small contact pads labeled plus and minus. To connect the LED to the driver, two connections are made by soldering wires to the plus- and minus-labeled contact pad on the PCB. Preassembled LED options also are on the market but these options are much more expensive. In general, it is more cost-effective to invest in a digital soldering station rather than buying pre-assembled LEDs from third party suppliers. There are several good soldering tutorials on the Internet, and we only provide a short description here.

**Fig 5:**
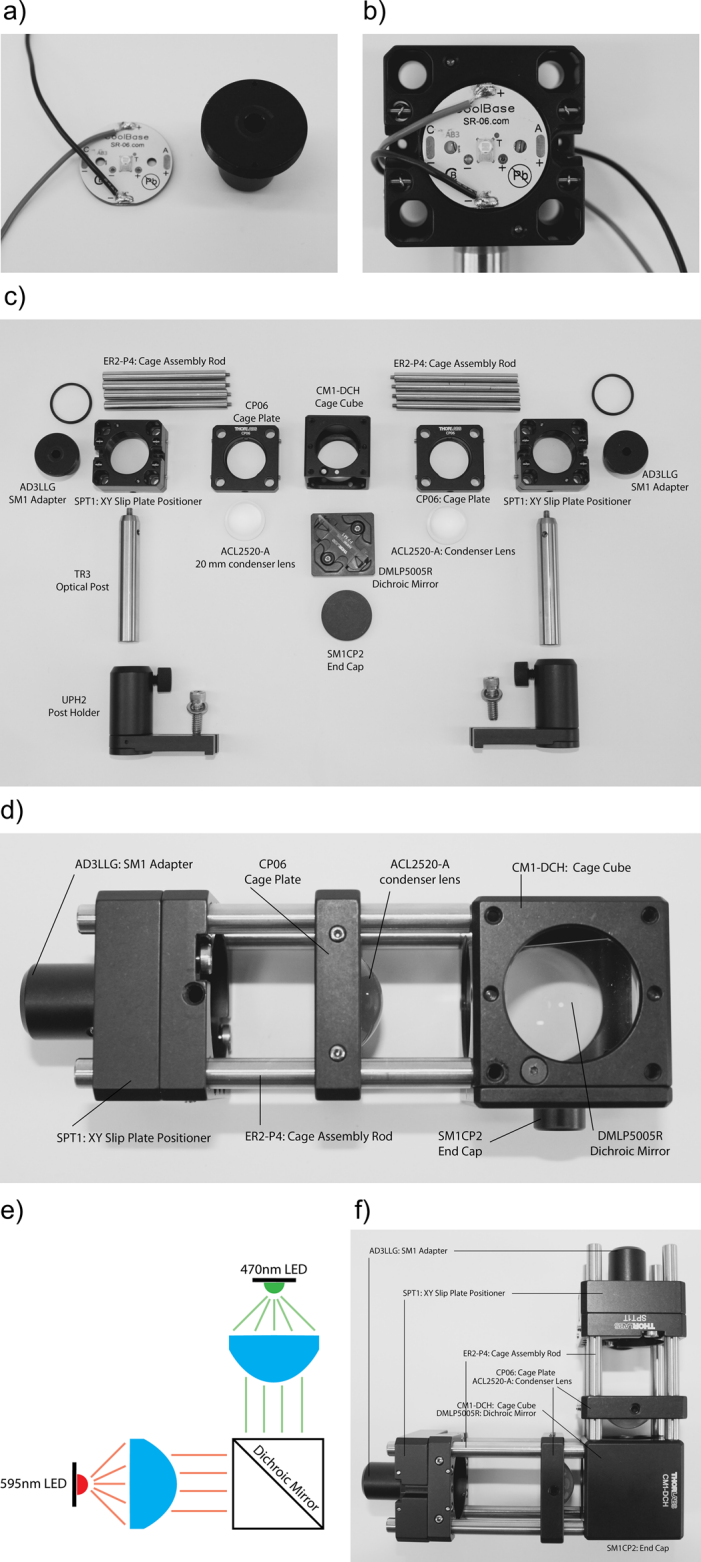
LED and optomechanical assembly. a) Luxeon LED on a 25 mm PCB with wires soldered onto them next to a LLG holder (Thorlabs AD3LLG), here used as a heatsink. b) Assembly consisting of a LED glued on top of an LLG holder and screwed into a xy slip plate (Thorlabs SPT1). c) Overview over the parts needed to assemble the LED illuminator structure. The orientation of the parts in the photograph roughly reflects the order of assembly. d) One arm of the illuminator attached to the dichroic filter cube. e) Schematic and f) photograph of the final assembly. Note that the coated-surface of dichroic mirror should face and reflect the longer wavelength source.

Before handling electronic components, the operator and ideally also the work surface should be properly grounded. An antistatic wrist wrap connected to a proper earth ground can protect expensive components from electrostatic discharge. Soldering should be done by first placing the LED PCB onto a nonflammable surface or clipping it into a soldering “helping hand” stand. Degreasing the soldering pad and wire with isopropyl alcohol can help to achieve a good solder joint. The soldering iron should be preheated to about 350°C, and the soldering tip brought into contact with the PCB. Next, a small amount of solder is applied such that the molten solder flows onto the preheated contact pad. Next, also the wire (we recommend 24 Gauge, silicone-coated wire) is coated with a thin layer of solder. Then the wire is brought into contact with the pad and the soldering iron tip is used to melt the solder on the pad such that the wire is covered. This technique ensures a good solder joint. Fig 5A depicts a Luxeon LED on a 25 mm PCB with wires soldered to the contact pads.

While LEDs are more efficient than arc bulbs, they produce a fair amount of heat. Accordingly, a heatsink is required to prevent unstable light output and reduced lifetime. We found it straightforward to integrate the heatsink into the optical assembly by gluing the PCB onto a 1” threaded LLG adapter (Thorlabs AD3LLG) (Fig 5A) as it provides a large aluminum body. In addition, this adapter is compatible with xy slip plates (Thorlabs SPT1) (Fig 5B), which are essential to align the LED. Alternatively, a 1” threaded plates can be used (e.g. Thorlabs SM1AD5).

We recommend using thermal adhesive, which is typically used to adhere computer chips to heatsinks (e.g. e.g. Arctic Alumina thermal adhesive), to attach the LED PCB to AD3LLG adaptor. It is important that the PCB is well centered before the adhesive hardens. LEDs on 25 mm round PCBs instead of star-shaped PCBs are easiest to center.

#### Optomechanical assembly

There are two main options to assemble the different optical elements into a rigid structure from “optical LEGO” parts. The first option is a tube system. The second is a “cage”-based system. The advantage of a tube-system is that the optical elements are protected from dust. The disadvantage is that it is harder to adjust the optical elements. For example, we found that minute changes in the position of the collimation lens have significant impact on evenness of illumination. Also, xy translation of the LEDs can be helpful in centering the LEDs to the field of view, which is not possible with a tube-system. For these reasons, we chose the cage-system. All parts used in this description are shown in Fig 5C and listed in S1 Table.

To assemble the structure, screw the AD3LLG adapter with the attached LED PCB into the SPT1 slip plate positioner and adjust the PCB orientation such that the exiting leads are not blocking the LED (Fig 5B). Using latex gloves and avoiding touching the optical surface, insert the 20 mm collimation lens (ACL2520-A) into the CP06 cage plate and tighten the tip screw gently. Again, using latex gloves and avoiding touching the optical surface, insert the dichroic mirror (DMLP505R) into the cube (CM1-DCH). Note the orientation of the dichroic mirror’s coated side in the cube. Take four 2” cage rods and screw them into the cube such that the lower wavelength LED is reflected off the coated surface. Slide the cage plate holding the collimation optics onto the cage with the convex side facing the cube. Then slide the slip plate containing the LED onto the rods (Fig 5D). Repeat for the second LED and attach it to the cube such that the light is directed towards the non-coated side of the dichroic mirror, combining the light of both LEDs (Fig 5E and 5F). Make sure to add a SM1CP2 end cap to the cube side that is not used as additional dust protection.

To connect the illuminator to the microscope illumination port, we recommend an adapter (e.g. Thorlabs SM1A26) that can be connected to the filter cube using a lens tube coupler (Thorlabs SM1T2).

#### LED drivers and microcontroller interfacing

**Fig 6:**
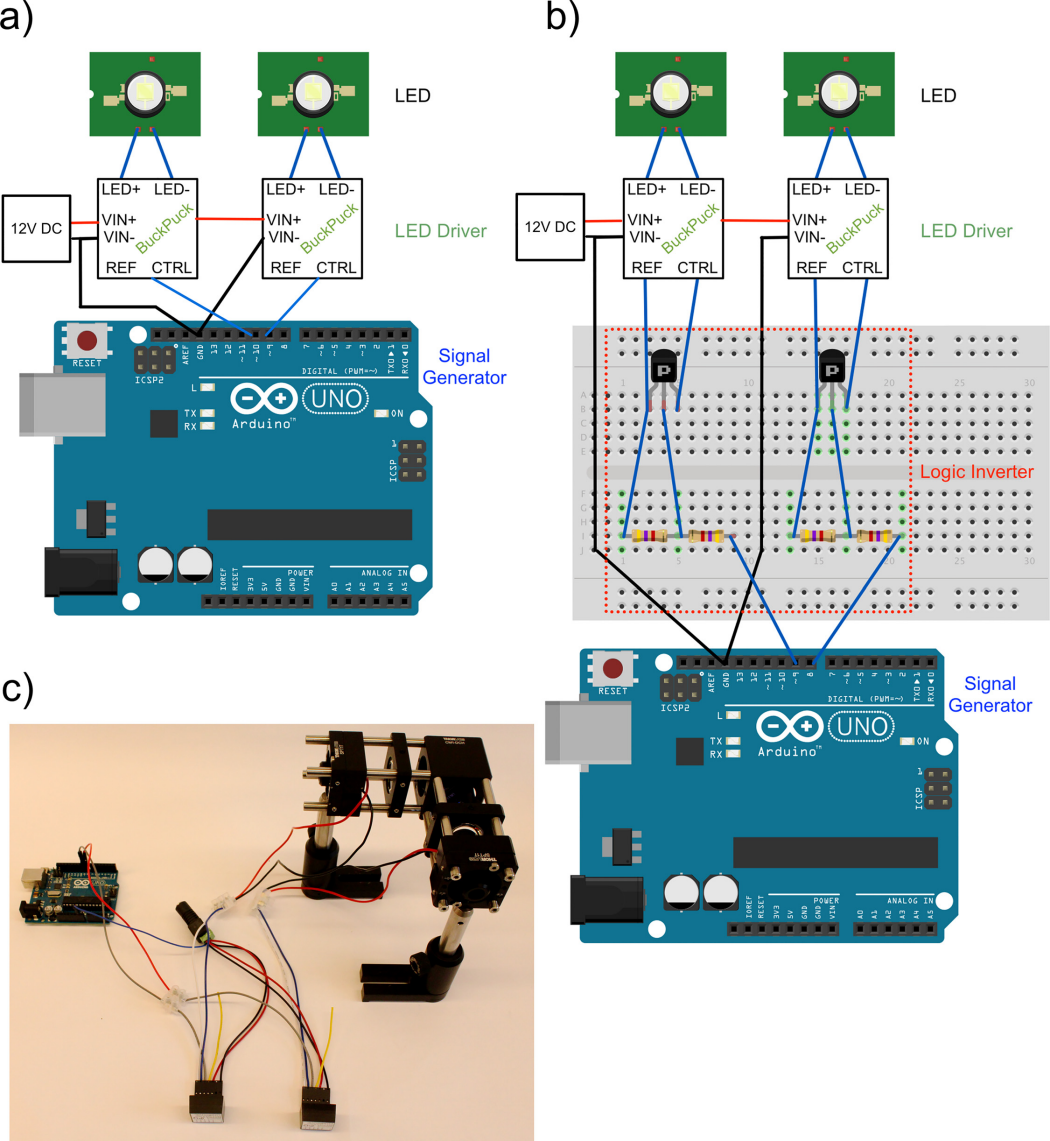
Electronic circuitry for LED drivers and microcontroller interfacing. a) Electronic wiring of the LEDs to drivers and the Arduino Uno microcontroller for LED-modulation without logic inverter and b) with logic inverter showing the circuitry with the additional components partially drawn with Fritzing [17]. Blue lines are signal connections, red lines are positive connections and black lines are ground connections essential to avoid different potentials between components. A 12V power supply (12V DC) of at least 3A is needed. The logic converter consists of 2N3906 PNP transistors and two 4.7 kohm resistors. c) Complete assembly with two LEDs mounted, two Buckpuck drivers with wiring harness and connectors wired to an Arduino Uno. The posts and post holder on the illuminator assembly are optional. The output port of the dichro cube can be connected to a microscope port adapter.

LEDs require a constant current source. There are many options, but we found that commercially available LED drivers are the easiest to use. We recommend the 1000 mA BuckPuck drivers (LEDdynamics) with a preassembled wiring harness and integrated potentiometer for current adjustment. This driver incorporates the driving electronics as well as a control unit that accepts a simple 5V signal to modulate the LED. However, its logic is reversed (5V signal at the Ctrl pin shuts the LED off) so either a logic inverter is needed or the logic of the signal generator has to be reversed. Fig 6A shows a wiring diagram without a logic inverter and Fig 6B shows a wiring diagrams with logic inverter (see also the corresponding circuit diagrams in the BuckPuck datasheet [16]). Both options will be discussed in more detail in the next paragraph. We recommend screw terminal blocks to connect the wiring harness to the wires soldered to the LED PCB. Fig 6C depicts the finished assembly without logic inverter. We recommend setting the current using a multimeter set between the driver’s plus pole and the LED plus pole. Then, turn the driver’s potentiometer up until the current reaches the desired current value. We recommend 1000 mA for the Luxeon Rebel blue (470 nm) and PC amber LEDs. Note that the connection between LED driver and LED should be kept short. It is advisable to protect the LED driver assembly with a plastic “project box” that can also be machined to hold BNC terminals (e.g. Thorlabs RBX-BNC) for the connection of Arduino and drivers with BNC cables. A 3D printed box would be another good alternative. We had success using a simple 12V 6A power supply, which is sold as a replacement for LCD computer monitors. However, a higher quality power supply might be needed if output stability is of utmost concern.

#### Computer interface

We recommend μManager [18] as imaging software because it is open-source, free, and allows the use of the low-cost Arduino Uno [19] platform as signal generator and triggering device. It is possible to use other software solutions, like Nikon NIS, but this requires costly upgrade packages to enable features like triggering, and rely on expensive DAQ boards for signal generation.

μManager can use pins 8–13 on the Arduino to control illumination channels. Wire the Arduino to the LED drivers according to Fig 6A or 6B, without or with logic inverter, respectively (circuit diagrams can also be found in the BuckPuck datasheet [16]). If no logic inverter is used, the logic of the Arduino during the μManager setup has to be reversed. If the logic inverter is used, we recommend testing the wiring on a breadboard first. Afterwards, the circuit can be soldered onto a standard circuit board.

Next, install the Arduino software from Arduino.cc. Download the Arduino adapter from the μManager website under device adapters and save the file as “AOTFcontroller.ino” in a new subfolder titled “AOTF controller” inside the main Arduino folder. If you are unable to download the Arduino driver (called “firmware source code”), copy-paste the text in the driver link into a notepad file and save the file as “ATOFcontroller.ino” on the desktop. Next, move this file into a subfolder titled “AOTF controller” within the main Arduino folder. Connect the Arduino via USB. Open the Arduino software and select the right board type (Arduino Uno) and the right serial port. Open the AOTFcontroller.ino file and upload it to the Arduino. Next, open μManager, open the hardware configuration wizard under Tools and create a new configuration for “Arduino” from the list of device adapters. Scan for the Arduino and select the COM port number in the value row. Next, leave the default Arduino Hub Logic to “normal” if you use a logic inverter or, set it to “inverted” if no inverter is used and click “Scan” to find the hardware. Also select all options except “DAC” in Peripheral devices setup. Next leave Arduino input at “all” and pull-up-resistor “on.” After installing the Arduino, save your presets as a new configuration file. Similarly, download the appropriate camera driver, and install it in μManager. Back in the main window, configure a new group with the name “LED channels”. Select Arduino Switch State. Next, select this group from list and click plus button next to Presets. Add first Preset and name it “470 nm”. Set Preset value in Arduino Switch state to 1 (Pin 8 on). Repeat and add second with name it “570 nm”. Set switch state to 2 (Pin 9 on). You should now be able to turn the LEDs on/off through μManager.

#### Collimating and alignment

After assembling the full unit (Fig 6C), the LEDs must be collimated and aligned. To collimate the LEDs, slide the slip plate to the end of the rods and fix them with the four setscrews. Next, point the LED at a distant wall and switch on only this LED (e.g. by detaching one driver from its harness). Slide the collimator lens back and forth until an image of the LED is formed at the wall. Note that the further the wall is away, the better the collimation will be. Fix the position by tightening the setscrews. Repeat with the second LED. Next, switch on both LEDs and point them on a nearby surface. Use the slip plates to overlap both beams onto each other.

It can be also helpful to use a fluorescent plastic slide (e.g. Chroma 92001) and adjust both collimation and alignment more finely while the unit is attached to the microscope. This is especially advantageous to maximize brightness and field homogeneity.

#### Triggering

Triggering, or hardware synchronization, is critical to achieve high-speed multi-channel switching necessary for imaging rapid and dynamic biological processes. The rate of image acquisition often is limited by the inefficient communication between the various components of the microscope. In addition, general-purpose computer operating systems do not function in real time, which can result in delays between images. Both factors are problematic for rapid live cell microscopy. Hardware synchronization, or triggering, can bypass these limitations and reduce phototoxicity by making the most efficient use of the exposure time [20]. Here, the imaging software sends a list of instructions at the beginning of the acquisition. The actual synchronization between the components is then done without interference from the main computer. Hardware triggering is often expensive because of specialized hardware and software needed. However, μManager supports triggering with an Arduino, which makes hardware synchronization available at much reduced cost. In general, also PIC controllers or logic boards with optoelectronic isolators are possible options, however we will focus on the Arduino here.

Triggering requires the use of a logic inverter between Arduino and LED driver, as depicted in Fig 6B. Additionally, a scientific camera that can generate a 5V pulse during exposure is essential. This “fire pin” is connected to pin 2 on the Arduino and a ground connection is established between the camera and the microcontroller.

In μManager, the property “Switch sequence” will then enable triggered “sequencing” mode. This setup allowed us to maximize dead times between frames to the technical limitations of the used camera (e.g. 3 ms for an Andor iXon3 897).

#### Dimming

Some applications require dimming of LED intensity. A simple solution is to place neutral density filters in front of the LEDs. However, if intensities need to be changed regularly, electronic dimming is advantageous.

**Fig 7:**
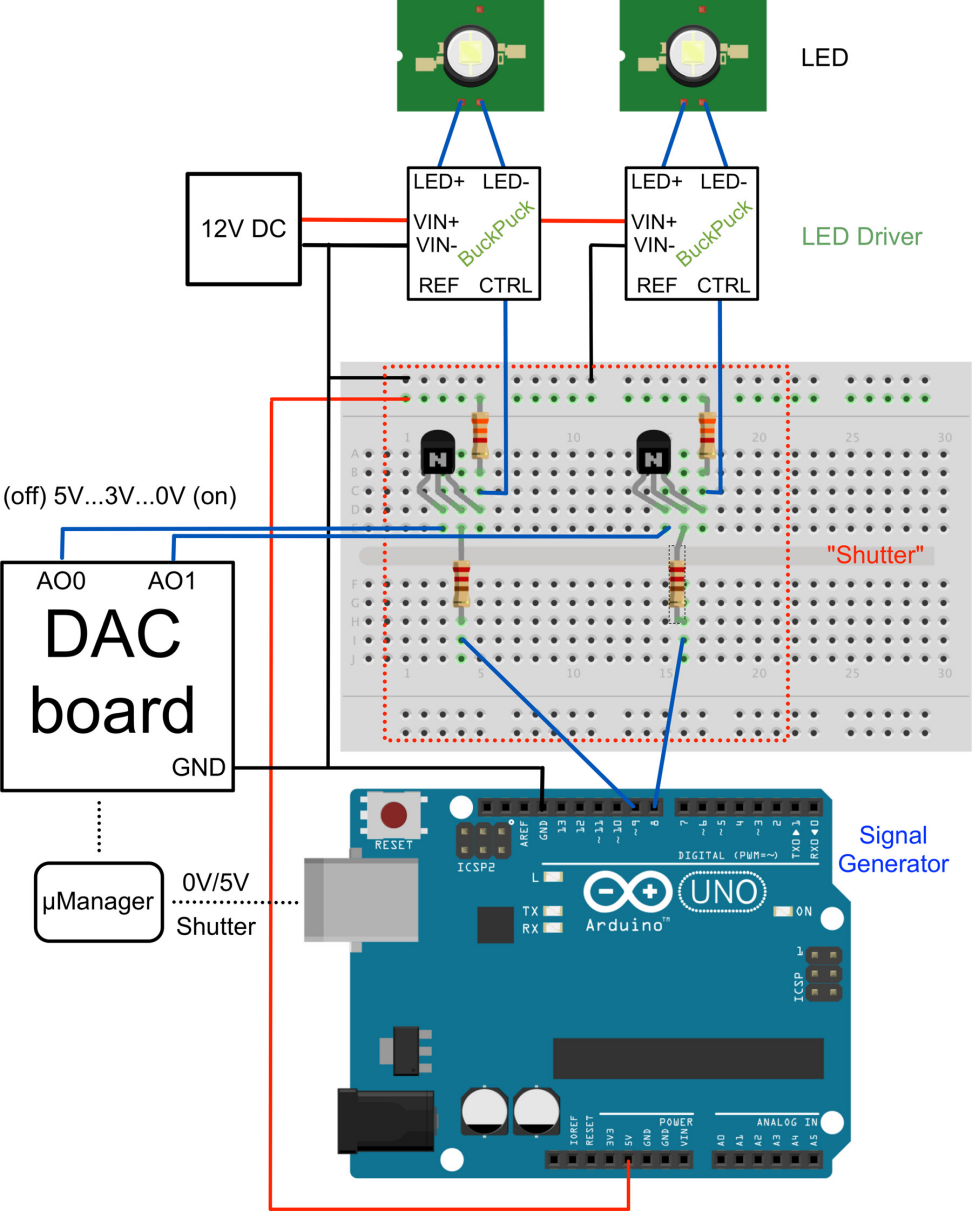
Dimming circuitry allows dimming through μManager. If brightness modulation through μManager is needed, an alternative circuit can be used. Here, a digital to analog converter (DAC) is used to generate voltage levels between 0 to 5 V. As the voltage sensing circuitry in the BuckPuck driver is reversed, 0 V corresponds to LED on and 5V to LED off. The dimming response curve is however not linear and can be found in the BuckPuck data sheet [16]. As the DAC board does not allow triggering through μManager, an Arduino is used instead and its signal is used to switch the DAC signal on and off through a simple 2N3904 NPN transistor. A 330 ohm resistor is used to protect the transistors gate (middle pin) and a 3.3 kohm or higher resistor is used as a pull-up resistor making sure that the LED shuts off when the Arduino signal is off. Blue lines show signal connections and black lines ground connections. Red lines show a 5 V line used for the pull-up resistors. A 12V power supply (12V DC) of at least 3A is needed. The schematic was partially made with Fritzing [17].

There are two possibilities to achieve electronic dimming: either the current flowing through the LED is adjusted (current regulation) or the LED is switched on and off with microsecond speed (pulse-width modulation). Current modulation can subtly change the color spectrum of LEDs, while PWM modulation can induce artifacts at very short camera exposure rates. Currently, μManager does not support either method directly through the Arduino hardware. However, current modulation can be implemented using a USB DAQ board (e.g. NI-USB 6008) (Fig 7). No logic inverter is needed. Basically μManager generates a voltage between 5 V (LED off) and 0 V (LED on) through the DAQ boards analog output channels. The BuckPuck driver has a voltage sensing circuit connected to its Ctrl pin (see its datasheet [16] for a response curve) such that the DAQ-board output essentially controls the LED brightness. To make this method compatible with triggering, a transistor must be introduced between the DAQ board pin and the LED driver. A pull-up resistor is required to turn the LED off when the transistor is not triggered (Fig 7). The Arduino output is then connected to the transistor gate and modulates the DAQ board.

Alternatively, dimming can be achieved by PWM modulation. However, μManager currently does not support PWM with the Arduino. Therefore, the DAQ output is fed into the ADC (analog to digital converter) of a second Arduino. This Arduino converts the measured voltage into a PWM signal, which is then used to control the LED driver.

Both solutions are not ideal and we urge our readers to join the open source community developing μManager to make LED dimming more straightforward. One possibility would be to implement direct PWM modulation into the Arduino device adapter.

### Multicolor single particle tracking in neurons

To test the performance of our LED illuminator in live cell imaging, we first seeded embryonic rat superior cervical ganglion (SCG) neurons in modified Campenot tri-chambers, as previously described [4]. After approximately 3 weeks in culture, we infected the cell bodies with a pseudorabies virus mutant (PRV 137) expressing a gM-EGFP fusion protein (as a virion envelope tag) and an mRFP-VP26 fusion protein (as a capsid tag) [5]. At 10 hours post-infection, we visualized dual-color virus particles moving rapidly in axons. To our surprise, blue LED illumination was so bright that we had to introduce an 10x neutral density filter (10% transmission) in front of the LED collimator because the intense light not only bleached the fluorescence of the virus particles within seconds, but total axonal trafficking was inhibited, possibly due to phototoxic effects. In comparison, the amber LED illumination did not induce any obvious phototoxic effects, and provided sufficient intensity to image particles at 50 ms per frame on an EMCCD camera with EM gain enabled.

With metal halide lamp illumination, an 8x neutral density filter was required (10% transmission), as blue excitation light was too intense. But, because the metal halide lamp is a combined white light source, the neutral density filter also reduced the amber illumination spectrum, effectively reducing the output of this channel similar to that produced by the amber LED intensity. The metal halide lamp, therefore, had no advantage in the amber spectral region compared to our LED source.

**Fig 8:**
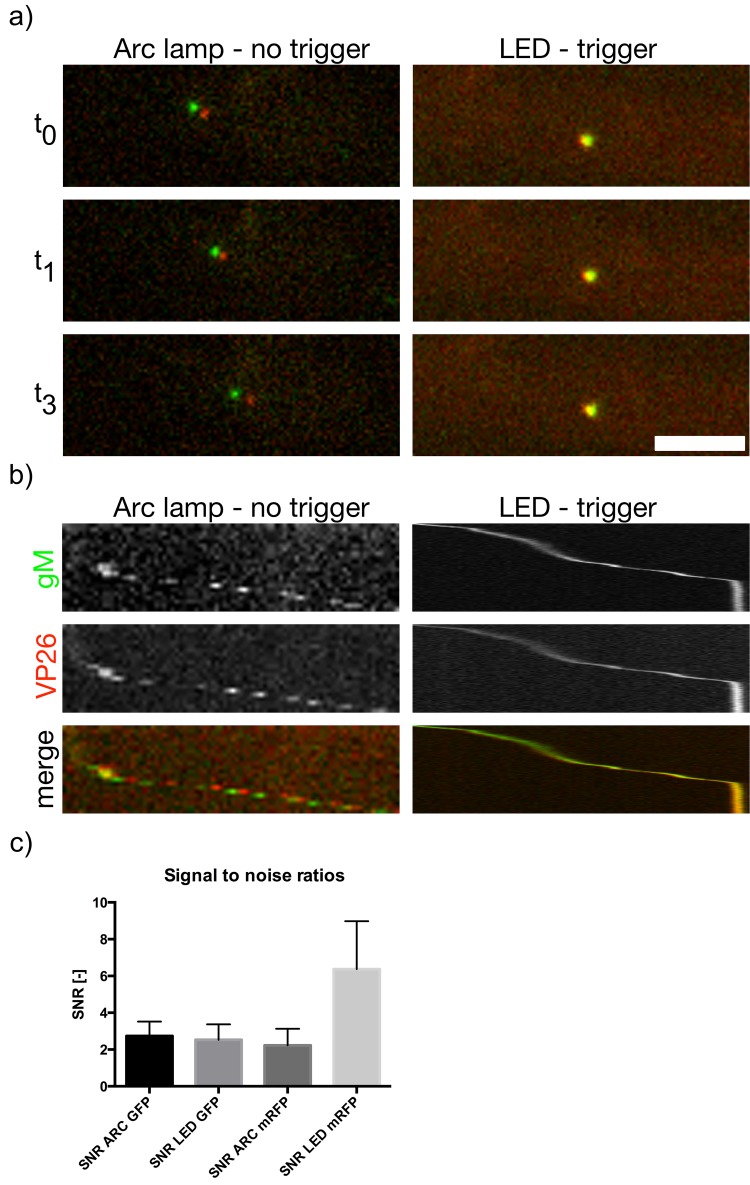
Triggering allows high-speed multi-channel live-imaging of axonal transport. Embryonic superior cervical ganglion (SCG) neurons were cultured in tri-chambers [4] and neurons in the soma-compartment were infected with PRV 137 expressing gM-EGFP (gM, green) and mRFP-VP26 (VP26, red). 10 hours post infection viral particle trafficking was imaged in axons penetrating into the middle and neuron compartment using non-triggered Arc lamp illumination (Arc lamp–no trigger) or triggered LED illumination (LED–trigger) (for details see Materials and Methods), a) Three consecutive stills of a representative track for each mode with roughly the same length (≈14 sec) and average particle speed of ≈1.5μm/sec are shown. The time between consecutive dual-color images is ~106 ms for LED illumination and 625 ms for Arc lamp illumination. b) The same tracks as in a) shown as a Kymograph. As triggered LED illumination allows much higher frame rates, particle positions with LED illumination almost overlap in both channels while they do not for non-triggered Arc lamp illumination. c) Signal-to-noise ratio (SNR) calculated for each imaging condition. Scale bar indicates 5 μm.

The metal halide lamp illumination was much slower in image acquisition. It took about 625 ms to acquire both channels with 50 ms exposure each (2x 50 ms plus 525 ms overhead, resulting in 1.6 dual color frames per second (or 3.2 Hz). due to mechanical filter switching and the lack of triggering capability. In contrast triggered LED illumination was able to record a dual-color image in 106 ms (2x 50 ms per frame plus 6 ms overhead) resulting in about 9.5 dual color frames per second (or 19 Hz). The lack of speed when using Arc lamp illumination was visible when recording fast axonal transport of viral particles. As shown in Fig 8A as consecutive stills and in Fig 8B as kymographs, the lag time during filter switching with metal halide lamp illumination led to different particle positions for each color channel, while signals mostly overlapped with triggered LED illumination (see also S1 and S2 Files for the raw data). The signal-to-noise ratios, which are crucial for localization precision, were comparable between imaging conditions (Fig 8C, for raw data see S6 Table).

It is important to note that the acquisition speed when using triggered LED illumination is not limited to 50 ms per channel. Instead, frame rate is limited by the brightness of the sample and its sensitivity to photo-damage as well as the cameras technical limitations. An appropriate frame-rate should be chosen such that the samples dynamics are not oversampled to avoid phototoxic effects. In general, the maximal required frame rate can be calculated in single particle experiments by first determining what localization accuracy can be expected at a certain signal-to-noise level. Assuming a localization accuracy of +/- 40 nm and a maximal particle speed of 2 μm per second (2 nm/ms), a maximal exposure of 20 ms for both channels combined is appropriate (40 nm/2 nm ms^-1^ = 20 ms) to acquire the maximal amount of data without oversampling. In our imaging example, this exposure would have been possible by cropping the camera field of view, which increases the maximal frame rate of the camera. But, faster exposure also decreases the signal-to-noise ratio. Therefore, small residual differences in particle positions (as shown in Fig 8A, LED illumination) have to be weighted against signal strength.

In conclusion, triggered LED illumination was much faster than non-triggered metal halide lamp illumination using filter wheels. While metal halide lamp illumination is theoretically brighter than LED illumination in the amber spectrum, the need to attenuate the blue spectrum in sensitive live cell experiments essentially negates this advantage because the needed neutral density filter simultaneously attenuates the amber spectrum to a level that is comparable the LED sources.

## Discussion

LEDs have become a viable option for fluorescence microscopy. They cost less than replacement arc lamp bulbs, have much longer lifespans, produce less heat, eliminate noise, and are mercury-free [21].

While there are several excellent reports that review and show the great utility of LED for microscopy [6–8], single color live cell microscopy [22], and FLIM and FRET microscopy [8], a practical guide and introduction into the use of LEDs for rapid live cell microscopy has been lacking. Here we evaluate several design considerations and demonstrate how to build a complete illuminator. We calculated the total cost for this system including software integration to about $3000 USD at the time of writing. This cost is possible largely due to availability of the open source software μManager [23] and its excellent Arduino device adapter, written by Nico Stuurman. Similar imaging solutions from a commercial source can be much more expensive.

While comparing LED outputs, we found that the blue LED illumination spectrum already is better than that produced by a typical metal halide lamp, while LEDs in the amber spectrum are about 6-fold dimmer with our specific filter set. Better matching filter sets, especially in the green-yellow-amber region might reduce that difference.

Most importantly, Haitz’s law predicts an exponential growth in LED efficiency such that future LED technology will be the method of choice. As our design is open and self-built, future LED technology can be integrated into existing platforms easily for negligible upgrade costs.

Advances in LED technology might also lead to smaller chip size, which would be advantageous as it reduces the problem of etendue. Another way to increase the amount of light is optimized optical elements to collect and guide more of the produced light to the objective. Some options include parabolic reflectors coupled in conjunction with liquid light guides, as are used for arc lamps, or compound parabolic concentrators, light pipes, and microlens arrays [24].

White light LED “light engines” are already brighter than arc lamps over most of the spectrum [21]. However, these solutions do not make use of the most important advantage of LEDs: their narrow spectral range. Therefore, mechanical filter wheels are still required, which slows down live cell imaging, and can lead to pixel shifts between channels. Commercial LED sources with separately modulatable channels exist, but are often quite expensive.

By comparing our open LED illuminator design with a metal halide lamp while imaging neurons, we found that LED illumination intensity in the amber region was well-suited for imaging single viral particles, while blue illumination required attenuation. The separated LED emitters facilitated dimming of individual channels, while Arc lamp illumination required general dimming (although switching of ND filters for Arc lamp illumination would have been possible using a costly third filter wheel). Therefore, our illuminator with today’s LED technology and simple collimating optics already outcompetes metal halide lamps both in speed and versatility. We demonstrate this point by using the system to image rapidly moving, single virus particles in neurons.

As LED technology is rapidly evolving, future developments are likely to lead to more interesting solutions. For example, the combination of several very small LED emitters of different colors into one package might eliminate the need of dichroic filters provided that the distance between the separate chips and the size of the resulting assembly is small enough. This advance could be combined with a design in which the LED emitter is integrated into the fluorescence filter cube [25]. Alternatively, a light pipe could be used to mix the colors. We tested this idea with a Quad-emitter Luxeon Rebel assembly (Luxeonstar Rebel Quad), but the emitters spacing of about 14mm from center to center was too large to mix the light output efficiently to guide it into the objective with a standard 20 mm aperture light pipe (Edmunds optics). However the new Luxeon Z LEDs can now be combined onto dense multi-emitter arrays that might be small enough to be coupled directly into a liquid light guide or light pipe for further homogenization.

In conclusion, we demonstrate the ease of building a simple and efficient research grade LED illuminator from inexpensive stock parts, even with minimal experience in optics or electronics. Integration with the open source software μManager allows hardware triggering. We showed that this self-built system allows the imaging of single virus particle dynamics in living axons and will be useful to image the dynamics of other cellular processes.

## Supporting Information

S1 Table Parts list. All parts and tools used in this work are listed. Parts can be substituted from other vendors and are only suggestions. Prices should be taken as guidelines to estimate total cost at time of publication and may change over time.
(XLSX)

S2 Table LED intensity measurements. Raw mean data used to prepare Fig 3A. See the materials and methods section for details.
(XLSX)

S3 Table LED intensity measurements. Raw mean data used to prepare Fig 3B. See the materials and methods section for details.
(XLSX)

S4 Table Tested LEDs. List of all LEDs tested as well as their tested drive currents and viewing angles.
(XLSX)

S5 Table Field uniformity measurements. Raw intensity data used to prepare Fig 4C. See the materials and methods section for details.
(XLSX)

S6 Table Signal to noise ratios for Arc and LED illumination. Raw values used to prepare Fig 8C.
(XLSX)

S1 File Non-triggered Arc lamp illumination of axonal transport. Raw image data used to prepare Fig 8. Image stack can be viewed in Fiji [3].
(TIF)

S2 File Triggered LED illumination of axonal transport. Raw image data used to prepare Fig 8. Image stack can be viewed in Fiji [3].
(TIF)
