# Supplementary figures and images for: Open LED Illuminator: A Simple and Inexpensive LED Illuminator for Fast Multicolor Particle Tracking in Neurons
Source: PLoS One. 2015 Nov 23;10(11):e0143547. doi: 10.1371/journal.pone.0143547 (PMC4658086; doi:10.1371/journal.pone.0143547)

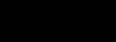

Supplement: S1 File — Raw image data used to prepare Fig 8. Image stack can be viewed in Fiji [3]. (TIF) [file pone.0143547.s007.tif]

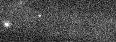

Supplement: S2 File — Raw image data used to prepare Fig 8. Image stack can be viewed in Fiji [3]. (TIF) [file pone.0143547.s008.tif]
